# Shotgun proteomic analysis of mulberry dwarf phytoplasma

**DOI:** 10.1186/1477-5956-8-20

**Published:** 2010-04-08

**Authors:** Xianling Ji, Yingping Gai, Baoyun Lu, Chengchao Zheng, Zhimei Mu

**Affiliations:** 1 College of Forestry, Shandong Agricultural University, Tai'an, Shandong, 271018, PR China; 2 State Key Laboratory of Crop Biology, Shandong Agricultural University, Tai'an, Shandong, 271018, PR China

## Abstract

**Background:**

Mulberry dwarf (MD), which is caused by phytoplasma, is one of the most serious infectious diseases of mulberry. Phytoplasmas have been associated with diseases in several hundred plant species. The inability to culture phytoplasmas *in vitro *has hindered their characterization at the molecular level. Though the complete genomes of two phytoplasmas have been published, little information has been obtained about the proteome of phytoplasma. Therefore, the proteomic information of phytoplasmas would be useful to elucidate the functional mechanisms of phytoplasma in many biological processes.

**Results:**

MD phytoplasmas, which belong to the 16SrI-B subgroup based on the 16S DNA analysis, were purified from infected tissues using a combination of differential centrifugation and density gradient centrifugation. The expressed proteome of phytoplasma was surveyed by one-dimensional SDS-PAGE and nanocapillary liquid chromatography-tandem mass spectrometry. A total of 209 phytoplasma proteins were unambiguously assigned, including the proteins with the functions of amino acid biosynthesis, cell envelope, cellular processes, energy metabolism, nucleosides and nucleotide metabolism, replication, transcription, translation, transport and binding as well as the proteins with other functions. In addition to these known function proteins, 63 proteins were annotated as hypothetical or conserved hypothetical proteins.

**Conclusions:**

Taken together, a total of 209 phytoplasma proteins have been experimentally verified, representing the most extensive survey of any phytoplasma proteome to date. This study provided a valuable dataset of phytoplasma proteins, and a better understanding of the energy metabolism and virulence mechanisms of MD phytoplasma.

## Background

The mulberry tree, whose leaves are the chief food for mulberry silkworm (*Bombyx mori *L.), is a perennial woody plant of considerable economic importance and has long been cultivated for sericulture. Mulberry trees are often affected by a number of diseases prevalent throughout their life cycles in different agroclimatic zones. Among all the diseases, mulberry dwarf (MD) is one of the most serious infectious diseases [1]. The causal pathogen of this disease, MD phytoplasma, was observed in the sieve tubes of plants affected for the first time in 1967 [2]. Phytoplasmas infect several hundred plant species worldwide, representing about 100 families including ornamental plants and many important crops [3]. They are morphologically and ultrastructurally resembled animal mycoplasmas [4], which are cell-wall-less prokaryotes in the class *Mollicutes *[5]. Due to inability in culturing phytoplasmas *in vitro*, our knowledge about the underlying molecular mechanisms for the determination of factors involved in their pathogenicity and the symptoms evoked in the host plants is limited [5]. The complete genome of the onion yellows phytoplasma and '*Candidatus *phytoplasma australiense' have recently been published [6-8]. Although gene prediction programs have become more accurate and sensitive, it is still difficult to predict genes accurately from genomic data when the genes are small or have little homology to other known genes. Therefore, it is becoming increasingly important to annotate the genomes, and to distinguish between authentic genes and pseudo-genes. Verification of a gene product by proteomic methods is an important first step in 'annotating the genome' [9]. Analysis of proteins provides more reliable evidence for existence and function of predicted proteins. Although the study of phytoplasma proteins will benefit greatly from the availabe genome sequence, there are still a great many which remain to be identified. To better understand the functional mechanisms of phytoplasma in many biological processes, it is necessary to characterize the expressed proteome of phytoplasma. With the development of proteomics, more and more proteomics techniques (such as two-dimensional polyacrylamide gel electrophoresis (2-DE), matrix-assisted laser desorption ionisation-time-of-flight mass spectrometry, liquid chromatography tandem mass spectrometry, and surface-enhanced laser desorption/inionation-time of flight-mass spectra) are used to profile proteins [10]. Shotgun proteomics is a method of identifying proteins in complex mixtures using a combination of high performance liquid chromatography combined with mass spectrometry [11]. Compared with traditional proteomic methods such as 2-DE, shotgun proteomics serves as a powerful tool to separate and identify proteins from complex protein mixtures and possesses the virtues of high efficiency, time, and labor saving [12].

In this study, we performed nanocapillary liquid chromatography-tandem mass spectrometry (GeLC-MS/MS) to characterize the expressed proteome of mulberry dwarf phytoplasmas by using shotgun proteomics. This research provides not only a technique to study the phytoplasma proteome, but also a valuable dataset of phytoplasma proteins, thus providing better understanding of the functional mechanisms of phytoplasma in many biological processes.

## Results

### MD symptoms and MD phytoplasma phylogenetic analysis

**Figure 1:**
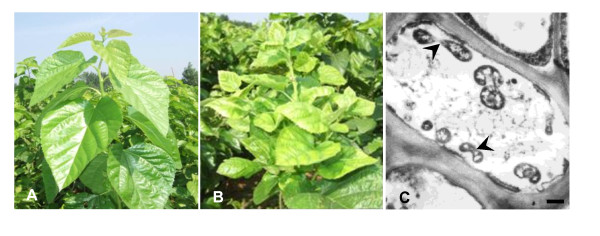
**Mulberry dwarf disease symptoms and mulberry dwarf phytoplasma cells in symptomatic area of the infected leaf**. A: Healthy mulberry tree; B: Mulberry dwarf disease tree; C: Mulberry dwarf phytoplasma cells in the infected leaf. Arrow indicates a typical mulberry dwarf phytoplasma cells. Bar 200 nm.

The infected mulberry plants developed typical MD symptoms including yellowing, chlorosis, phyllody, stunting, and witches' broom (Fig. 1B), when compared to healthy plants (Fig. 1A). Plants with MD symptoms were positive by PCR assay, whereas healthy ones were negative, with a diagnostic amplified fragment of 1097 bp containing the 16S rRNA gene (GenBank Accession No. EF532410). Phylogenetic analysis showed that the sequenced nucleotides were highly homologous to those of the 16SrI-B subgroup (aster yellows groups). In addition, the MD phytoplasmas were detected in the sieve tube of the infected phloem tissues by EM (Fig. 1C). The visualized cells were spherical or oval, from 50 nm to 200 nm in diameter, and with bilamina membrane. In contrast, these were not found in the healthy plant samples. No other pathogenic organisms, bacteria, virus, nor virus-like structures were observed.

### Isolation of MD phytoplasma from the infected tissues

**Figure 2:**
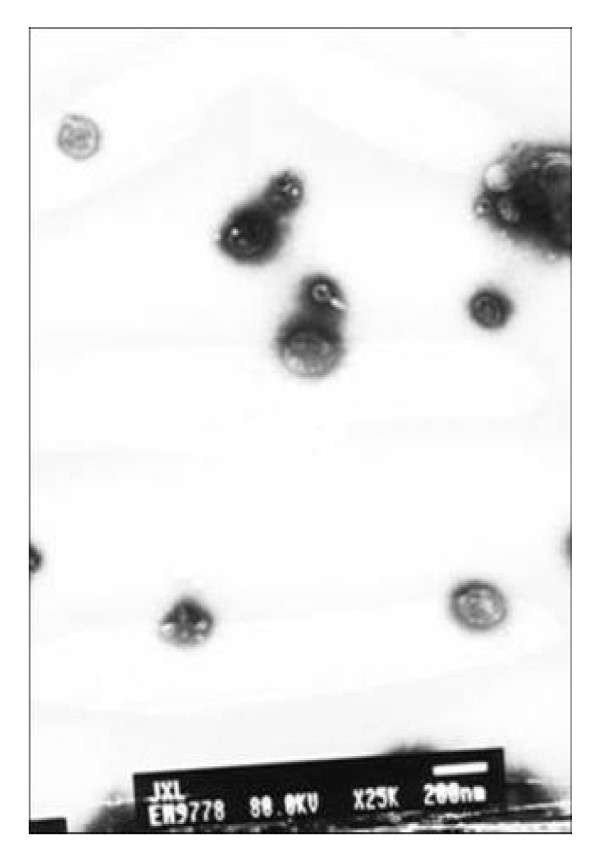
**Transmission electron micrographs of ultrathin sections of phytoplasma cells purified**. Bar 200 nm.

**Figure 3:**
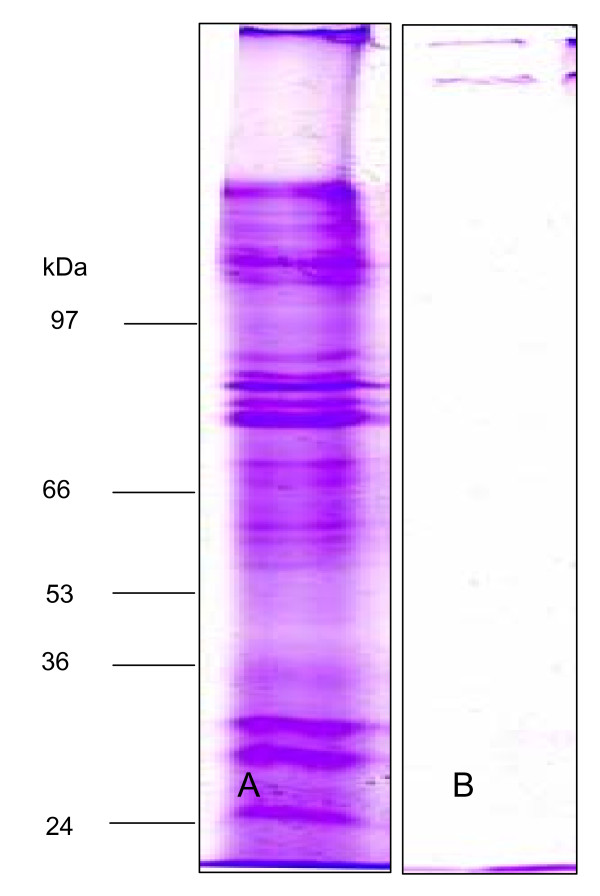
**Examination of the purified dwarf phytoplasma cell by one-dimensional SDS-PAGE**. A: Separation of phytoplasma proteins by one-dimensional SDS-PAGE; B: Separation of the healthy mulberry plant sample extracts from by one-dimensional SDS-PAGE. 150 μg of proteins was separated on 12% Bis-Tris gel and stained to allow protein identification.

The purified MD phytoplasmas were examined by TEM observation. The result showed that the visualized cells in the thin slices of purified samples were similar to those observed in the sieve tubes of infected plants. Most of the purified MD phytoplasmas were intact, and no organelles, plant cells and their debris were found (Fig. 2). To display and compare the extractions from infected and healthy mulberry plants, we carried out SDS-PAGE using the modified TCA/acetone extraction protocol in combination with CBB staining (Fig. 3). The result indicated that the purified sample from infected mulberry plants should be the MD phytoplasma. The purified sample was subjected to trypsin digestion and analyzed by GeLC-MS/MS. Only a few mulberry protein were identified (see Additional file 1), which might further validate the purification protocol.

### Identification and categorization of the MD phytoplasma protein

**Figure 4:**
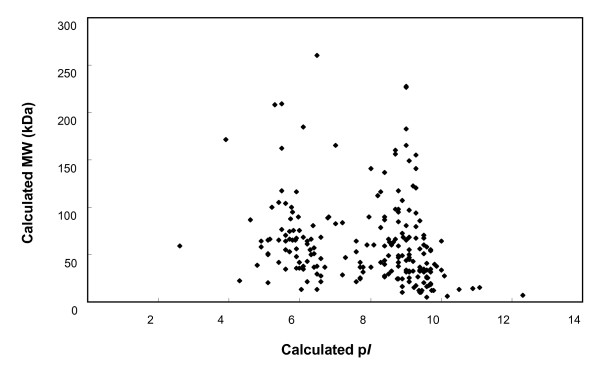
**The calculated p*I *of identified phytoplasma proteins plotted against their calculated molecular weight on a logarithmic scale**.

**Figure 5:**
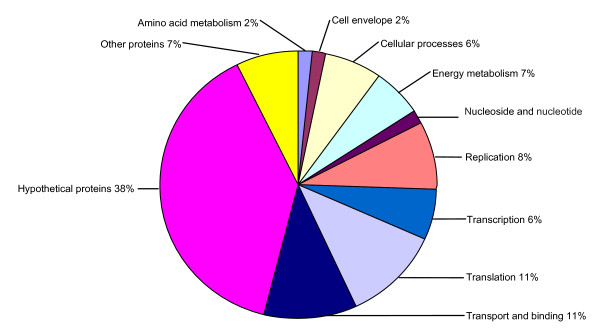
**The functional category distribution of the 209 identified proteins**.

The proteins presented in the gel bands were subjected to trypsin digestion and analyzed by GeLC-MS/MS. Using the criteria mentioned above, we identified 209 non-redundant proteins including membrane proteins, low abundance proteins, high molecular weight proteins, and proteins with extreme p*I*s (see Additional file 2 and Fig. 4). The most acidic and basic proteins identified by GeLC-MS/MS have p*I*s of 2.6 and 12.3, respectively (Fig. 4). These 209 identified proteins could be sorted into 11 categories according to their functions. The most abundant category was hypothetical proteins (38%), and transport and binding proteins (11%) and translation associated proteins (11%) were in the second place. Replication proteins (8%) were in the third place. Other functional categories included amino acid biosynthesis, cell envelope, cellular processes, energy metabolism, nucleoside and nucleotide metabolism, and transcription (Fig. 5).

## Discussion

### Phytoplasma energy metabolism

Phytoplasma, which inhabits nutrient-rich phloem tissue, has the presence of novel mechanism(s) for energy metabolism. In this study, 2 protein subunits (see Additional file 2, No. 28 and 29) encoded by genes of the ATPase operon were identified. However, phytoplasmas appear to have lost the genes for the ATPase during evolution, and were energy parasites unable to synthesize their own ATP [6]. So it is likely that these ATPase components identified are not involved in energy generation, but most likely candidates for generating the electrochemical gradient over the membrane [5]. This is similar to *Chlamydia trachomat*, which were thought to have lost the genes for the ATPase, but have several protein subunits encoded by the genes of ATPase operon [13].

**Figure 6:**
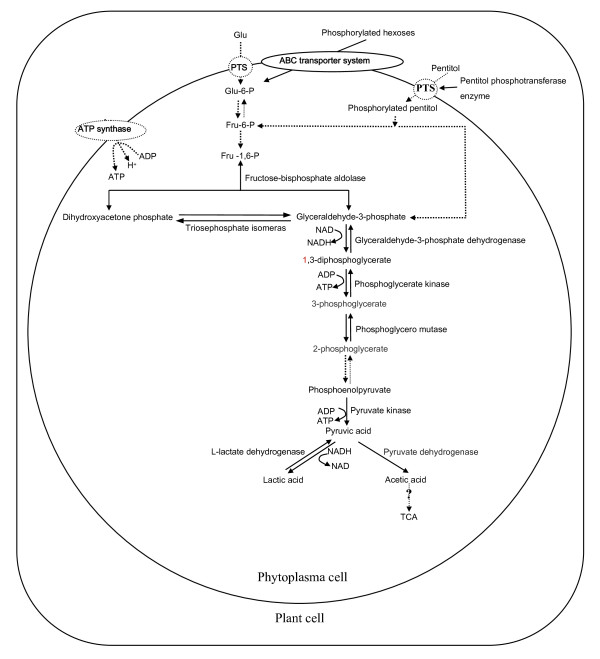
**Model for the energy metabolism of MD phytoplasma**.

Phytoplasmas have the genes encoding enzymes for glycolysis, suggesting that they possess a functional glycolysis pathway [14]. Seven enzymes associated with the glycolysis pathway, including glyceraldehyde-3-phosphate dehydrogenase, L-lactate dehydrogenase, phosphoglycerate kinase, pyruvate kinase, triosephosphate isomerase, fructose-bisphosphate aldolase and phosphoglycero mutase, are also present in our dataset (see Additional file 2, No. 26, 27, 30, 32, 34, 36, and 38). Phytoplasmas have no phosphotransferase system (PTS) to import sugars and to generate glucose-6-phosphate to feed the glycolysis pathway [7]. But the pentitol phosphotransferase enzyme, one of the PTS components, was found in our dataset (see Additional file 2, No. 35). It is not clear whether the enzyme has the function to import pentitol into the cell to feed glycolysis or it is just the left part of the structural genes which are not completely lost in evolutionary process. Our proteomic analysis supports the concept that phytoplasmas depend on the uptake of phosphorylated hexoses for their carbon source to enter the glycolytic pathway, because some kinds of ABC transporters (see Additional file 2, No. 104-122), which can import phosphorylated hexoses from the cytoplasm of host, were identified. But the conversion of these imported sugars to glucose-6-phosphate for glycolysis were not clear and the enzymes required for the conversion were not found in the phytoplasma genomes and our data, indicating that the glycolysis pathway in phytoplasma may be a concise one. In addition to the components of glycolytic pathways, the pyruvate dehydrogenase (see Additional file 2, No. 33) which is the key linker reaction enzyme of the glycolysis pathway and the citric-acid (TCA) cycle was found. Although genetic evidence shows that the TCA cycle is incomplete in phytoplasma, these findings support the idea that a modified pathway operates [6]. Therefore, the phytoplasmasare are capable of producing their own energy, but most likely depend mainly on the glycolytic pathway. The model for the energy metabolism of MD phytoplasma has been provided (Fig. 6).

### Phytoplasma virulence

Plant pathogenic bacteria possess different types of pathogenicity genes [15]. However, the phytoplasma genome possesses no genes homologous to these known pathogenicity genes, suggesting it has novel mechanisms for virulence. There was one hemolysin-like protein gene identified as a possible virulence factor in phytoplasma genome [14]. Our proteomic data proved the existence of such a hemolysin-like protein (see Additional file 2, No. 24), speculating that it had the same function as the bacterial virulence factor. Phytoplasmas lack many genes which are considered to be essential for cell metabolism, suggesting that they may import a vast array of biological molecules from the host cytoplasm. This may result in the metabolic unbalance of the host cell and cause the disease symptoms. The phytoplasma genome contains many genes encoding transporter systems, such as ABC transporters, which aggressively import nutrients from host cells [14]. Our proteomic analysis proved the existence of these transporters (see Additional file 2, No. 104-122). Some proteins of ABC transporters are usually secreted lipoproteins that bind an external substrate of the cell and deliver the substrate to the ABC transporters and may also be involved in adherence to cell surfaces [7]. Furthermore, some ABC transporters can also secrete toxins into the host cells. Hence, these proteins of ABC transporters are possible virulence factors of phytoplasmas. Immunodominant proteins of pathogenic bacteria are likely candidates for host-pathogen interactions such as attachment to host cells, which could be a prerequisite for colonization and infection [5]. One immunodominant protein was recognized in our phytoplasma proteomic analysis (see Additional file 2, No. 8), which is also a possible virulence factor of phytoplasmas.

### Hypothetical proteins

Hypothetical or conserved hypothetical proteins were proteins that were predicted from nucleic acid sequences only and either had no homologues in the protein sequence databases, or were homologous to genomic sequences of unknown functions [16]. These proteins are of interest as candidates for gene disruption to reveal new biological functions, especially if the predicted proteins can be shown to be temporally produced or have a known biological activity. Totally 63 proteins designated as 'hypothetical' or 'conserved hypothetical' were detected, making this the most numerous group of proteins identified in this study (see Additional file 2, No. 130-192). The detection of peptides from the predicted proteins substantiates the existence of the latter and paves the way for more detailed studies to characterize their functions. For phytoplasma has the novel mechanism(s) for virulence and energy metabolism, these hypothetical proteins may possibly be responsible for virulence, intracellular survival and energy metabolism. Further analyses of these proteins using bioinformatics tools, gene expression and specific antibodies will likely yield new scientific insights into the adaptation lifestyle and pathogenicity of this important plant pathogen.

### Other proteins

Genome analysis showed that phytoplasmas lack most of the genes necessary to synthesize nucleotide, amino acid biosynthesis and fatty acid [6,7]. Our proteomic analysis supports these results. Only a few proteins related to this process were detected in our data. So the phytoplasmas probably cannot synthesize most of the metabolites assimilated from the host cells. Although phytoplasmas are bounded to host tissues, their genomes contain a very limited number of genes encoding proteins related to the cell envelope biogenesis, cell motility, and secretion [14]. There were also a limited number of proteins detected in our data related to these processes. This might support that the ancestor of the phytoplasma may have possessed a cell wall [14]. Though phytoplasmas can import small biological molecules from the surrounding host cytoplasm, they cannot import functional housekeeping enzymes outside their cells. Phytoplasmas have a greater proportion of proteins for DNA replication, transcription, translation, and other cellular processes, suggesting that they possess a functional replication, transcription and translation pathway which may be a concise one.

## Conclusions

In conclusion, MD disease is associated with phytoplasmas belonging to the 16SrI-B subgroup (aster yellows). A method for the purification of MD phytoplasmas from infected tissues was developed for proteomic analysis. The expressed proteome of phytoplasma was surveyed by using a shotgun strategy. Taken together, a total of 209 phytoplasma proteins were verified, representing the most extensive survey of any phytoplasma proteome to date. Analysis of MD phytoplasma proteome suggests that the phytoplasmas depended mainly on a glycolytic pathway for producing their own energy. In addition, the phytoplasmas might have a novel mechanism(s) for virulence, and many of the proteins, which are possible virulence factors, were verified. The methods and data in our study might provide a technique to study phytoplasma proteome and a better understanding of the functional mechanisms of phytoplasma in many biological processes.

## Methods

### Polymerase chain reaction (PCR) detection of MD phytoplasma and sequence analysis

Naturally infected and healthy mulberry samples were collected from plants in the same garden showing either well-developed MD symptoms or no MD symptoms. PCR analyses were performed as follows. Total DNA was extracted using the cetyltrimethyl-ammonium bromide method as previously described [17]. The 16S rDNA of the phytoplasma was amplified by PCR using a universal primer set as described before [18]. The produced DNA fragments were individually subcloned into pMD18-T vector and sequenced. The nucleotide sequences were analyzed using the DNAMAN (version 4.0) and GenBank databases.

### Transmission electron microscopy (TEM) detection of MD phytoplasma cells in infected leaf

Small pieces of tissue cut from diseased and healthy leaves were prefixed in 2.5% (w/v) glutaraldehyde, 0.1 M phosphate-buffered saline (PBS) (pH 7.3) for 2 h at 4°C, and postfixed in 1% (w/v) OsO_4_, 0.1 M phosphate-buffered saline (pH 7.3) for 1.5 h at 4°C before dehydration using an ethanol series, and embedding in Epon 812 resin (TAAB, Berkshire, UK). Ultrathin sections were cut with an ultramicrotome (LKB 4800; LKB Products, Bromma, Sweden) and observed with a transmission electron microscope (JEM 1010; JEOL, Tokyo, Japan) at 80 kV after staining with uranyl acetate and lead citrate.

### Mulberry dwarf phytoplasma purification

Soft vascular tissues from the diseased mulberry plants were used for MD phytoplasma purification, following the method as previously described [19-21] with some modifications. One hundred and twenty grams of fresh tissues were cut into pieces and blended in cold PBS (pH 7.0), then were disrupted in a food processor at low speed for 1 min. The disrupted tissues were rinsed through a 100 (100 mesh/inch) mesh screen, and the vascular tissues were retained. The retained vascular tissues were homogenized in ice-cold buffer (0.3 M glycin, 0.01 M MgCl_2_, 80 U gentamycin, pH 7.4) until no vascular fibers were discernible and then were squeezed through three layers of cheesecloth. The solution was blended with the enzymes (cellulose 1 mg/ml, hemicellulose 0.4 mg/ml, tartaric acid enzyme 1.5 mg/ml) and maintained at 35°C for 2 h. The precipitate was collected by centrifugation at 12 000 g for 1 h and resuspended with ice-cold buffer (0.3 M glycin, 0.01 M MgCl_2_, 0.3 M mannitol, 0.02 M Na_2_SO_4_, 80 U gentamicin, pH 7.4). The suspension was subjected to two cycles of differential centrifugation (5000 g for 15 min and 12 000 g for 1 h at 4°C). The pellets were resuspended with the same buffer and clarified by centrifugation at 2000 g for 5 min. The supernatant was loaded onto a discontinuous Percoll density gradient and centrifuged as described by Jiang and Chen [21]. After centrifugation, the pellet was collected from the fraction at the interphase between 30% and 60% Percoll layers, diluted with suspending medium (0.3 M D-mannitol and 20 mM MOPS, pH 7.0) and then centrifuged at 100 000 g for 3 h at 4°C to remove the Percoll from the suspension. Soft vascular tissues from the healthy mulberry trees as control were extracted as above. The final pellets were resuspended in PBS (pH 7.0) and examined by electron microscopy observation. Soft vascular tissues from the healthy mulberry plants were extracted samely, and the pellets obtained were used as control.

### Examination of the purified MD phytoplasma cells

The extractions obtained as above were first negatively stained with OsO_4 _for transmission electron microscopy using formvar coated copper grids, onto 10 μl of MD phytoplasma cell suspension. After 1 min, the excess liquid was wicked off with a piece of filter paper, and 10 μl of a 2% OsO_4 _solution was placed on the grid. After 1 min of staining of the sample with OsO_4_, the excess solution was wicked off, and the grid was allowed to air-dry. Grid was examined by transmission electron microscopy as described above.

### MD phytoplasma protein extraction and SDS-PAGE

The extractions collected above were homogenized by ultrasonication, and extracted by TCA-acetone (10% TCA in acetone containing 0.07% (v/v) β-mercaptoethanol, and incubation overnight at -20°C). After centrifugation (25 000 g for 1 h at 4°C), the pellet was washed twice with acetone containing 0.07% (v/v) β-mercaptoethanol (-20°C) and applied on a 12% Bis-Tris gel. The electrophoresis was run on the Ettan DALTII unit (GE Healthcare, Uppsala, Sweden) and the gels were visualized by Coomassie staining.

### GeLC-MS/MS shotgun analysis of MD phytoplasma proteome

After visualization by Coomassie staining, the whole gel lanes of MD phytoplasma protein as above were cut into four pieces of equal size and subjected to in-gel tryptic digestion as described by [22]. 1-D-LC MS/MS was performed using an LTQ linear IT mass spectrometer (Thermo, San Jose, CA, USA). The system was fitted with a C18 RP column (0.15 mm × 150 mm, BioBasic C18, Thermo Hypersil-Keystone). Mobile phase A (0.1% formic acid in water) and the mobile phase B (0.1% formic acid in 84% ACN) were selected. The tryptic peptide mixtures were eluted using a gradient of 4-50% B over 50 min, 50-100% B over 4 min and maintained at 100% B for 6 min. The LTQ linear IT mass spectrometer was set so that one full MS scan was followed by ten MS/MS scans on the ten most intense ions from the MS spectrum with the following Dynamic Exclusion™ settings: repeat count 2, repeat duration 30 s, exclusion duration 90 s [18]. Each sample was analyzed in triplicate.

### Protein identification

A forward protein database was extracted from UniProKB http://www.uniprot.org/ using keyword "*Mollicutes*" http://www.uniprot.org/uniprot/?query=Mollicutes&sort=score. There were 36, 627 proteins reported by UniProtKB. For each reported protein sequence in the forward *Mollicutes *protein database, a reversed protein sequence was generated using in-house developed software. All reversed protein sequences were then attached to the end of the forward *Mollicutes *protein database to construct an integrated *Mollicutes *protein database, which was then used for protein identification. Each raw instrument data were first converted into mzXML format. All mzXML files were then uploaded into Computational Proteomics Analysis System empowered with three database search engines, SEQUEST, Mascot, and X!Tandem. Parameters used for all three protein database search engines are as follows: parent peptide ion mass-to-charge variation 10 ppm, fragment ion mass variation 0.7 Dalton, maximum missed cleavage site of trypsin 1. PeptideProphet and ProteinProphet were used to assess the confidence of peptide and protein identification, respectively. We further employed false discovery rate (FDR) to control the peptide identifications based on the probability scores given by PeptideProphet. We set FDR ≤ 0.05 for every MS/MS dataset. Functional classifications were performed according to [19]. To see if any contaminating proteins are present, the protein data were searched against the viridiplantae database as described above.

## Competing interests

The authors declare that they have no competing interests.

## Authors' contributions

XL was responsible for design, sample preparations, PCR analysis, electron microscopy detection and protein purification, and contributed to write the manuscript. YP carried out GeLC-MS/MS shotgun analysis, identification of proteins obtained and also contributed to write the manuscript. BY carried out SDS-PAGE analysis. CC and ZM made substantial contributions to the study conception and design and critically revised the manuscript for intellectual content. All authors edited the manuscript and approved the final version.

## Supplementary Material

Additional file 1 **Mulberry dwarf phytoplasma proteins identified in this study.** M: Experimental molecular weight; C: Percentage of protein amino acid sequence coverage by the identified peptide Click here for file

Additional file 2 **Mulberry proteins identified in the purified sample from infected mulberry plants.** M: Experimental molecular weight; C: Percentage of protein amino acid sequence coverage by the identified peptide. Click here for file
