# Supplementary material for: Shotgun proteomic analysis of mulberry dwarf phytoplasma
Source: Proteome Sci. 2010 Apr 8;8:20. doi: 10.1186/1477-5956-8-20 (PMC2873370; doi:10.1186/1477-5956-8-20)
Supplement: Additional file 1 — Mulberry dwarf phytoplasma proteins identified in this study. M: Experimental molecular weight; C: Percentage of protein amino acid sequence coverage by the identified peptide [file 1477-5956-8-20-S1.DOC]

| No. | Protein name | The unique peptide used to identify the protein | Accession No. | M | pI | C |
| --- | --- | --- | --- | --- | --- | --- |
| 1 | Rubisco large subunit | R.LEDLRIPNAYIK.T | Q32625 | 51.6 | 6.0 | 3.01 |
| 2 | Sedoheptulose-1,7-bisphosphatase | R.DQVAAAMGIYGPR.T | P46283 | 42，4 | 6.17 | 3.56 |
| 3 | Rubisco activase | R.VPIIVTGNDFSTLYAPLIR.D | Q01587" | 45.7 | 7.57 | 5.08 |
| 4 | Maturase | R.YADNLLLGIVGSVELLIEIQK.R | O79414 | 71.6 | 10.15 | 3.12 |
| 5 | Unknown protein | R.TGKLIEVYRIWR.S | O22792 | 11.6 | 8.3 | 1.15 |
| 6 | Unknown protein | R.CSSCPTVATAPK.T | P18025 | 25.5 | 4.65 | 5.17 |
| 7 | Chloroplast precursor | K.LDKLHMTSGSVEIQQYDGR.V | P43310 | 73.2 | 5.89 | 1.88 |
| 8 | Polygalacturonase-inhibiting protein | K.SLISLDINHNK.I ! K.SLISLDLNHNK.I | Q05091 | 29.6 | 6.22 | 4.09 |
| 9 | PHYA4 photoreceptor | K.GIQM*SNNCTEHGFKETLYGDSLR.L | P33530 | 123.8 | 5.96 | 2.05 |
| 10 | RNA polymerase beta chain | R.KSSIVSFSLHK.D | Q06H07 | 96.6 | 9.66 | 1.30 |
| 11 | Putative far-red impaired response protein | K.DISNVRTTINK.E | Q25AC7 | 133.1 | 6.55 | 0.96 |

**Fig. 6**
