# Supplementary material for: Shotgun proteomic analysis of mulberry dwarf phytoplasma
Source: Proteome Sci. 2010 Apr 8;8:20. doi: 10.1186/1477-5956-8-20 (PMC2873370; doi:10.1186/1477-5956-8-20)
Supplement: Additional file 2 — Mulberry proteins identified in the purified sample from infected mulberry plants. M: Experimental molecular weight; C: Percentage of protein amino acid sequence coverage by the identified peptide. [file 1477-5956-8-20-S2.DOC]

| **No.** | **Category/protein name** | **The unique peptide used to identify the protein** | **Accession No.** | **M** | **p*I*** | **C** |
| --- | --- | --- | --- | --- | --- | --- |
|  | **Amino acid metabolism** |  |  |  |  |  |
| 1 | Dimethyladenosine transferase | K.FVKNSFK.Q | Q2NIH8 | 31.2 | 9.71 | 2.60 |
| 2 | Dipeptidase, putative | K.ELISKYFDQALNETMK.V | Q2SS68 | 50.8 | 5.1 | 3.56 |
| 3 | Asparagine synthetase AsnA | K.INFKYPDLVDK.L | Q6MRU7 | 38.0 | 6.1 | 3.37 |
| 4 | S-adenosylmethionine synthetase | R.AGM*LIKNQEFK.D | Q4A5F4 | 42.3 | 6.0 | 2.88 |
|  | **Cell envelope** |  |  |  |  |  |
| 5 | Putative integral membrane protein | K.SSVVTLNYNINNK.T | Q8EWK8 | 116.6 | 8.3 | 1.24 |
| 6 | p76 membrane protein precursor | K.EEVLPPLEEEKK.P | Q4A7M6 | 159.8 | 8.7 | 0.85 |
| 7 | Putative inner membrane protein | R.NINELLKDEQSLQALK.D | Q98R58 | 70.1 | 9.5 | 2.63 |
| 8 | Immunodominant protein | K.NQDQQKDDIKTK.A | Q9AIX3 | 32.8 | 9.2 | 4.18 |
|  | **Cellular processes** |  |  |  |  |  |
| 9 | Predicted hydrolase | K.IAYSNM'QFPKIEDYEK.A | Q6KH24 | 62.8 | 8.66 | 2.90 |
| 10 | Oligoendopeptidase F | K.YLKLCQLGGSQTFLNLLK.T | Q2NIT4 | 66.8 | 6.32 | 3.17 |
| 11 | Putative GTP-binding protein enga | K.PIILVVNKWDAVM*EKDER.S | Q14PC3 | 49.7 | 9.1 | 4.11 |
| 12 | GTP-binding protein lepA | K.KPVLYTGFYPIDTR.D | Q8GCP5 | 67.7 | 5.9 | 2.34 |
| 13 | GTP-binding protein lepA | K.TNGEIEFVSNPTM*M*PDR.S | Q4A5S3 | 68.3 | 6.1 | 2.81 |
| 14 | Translocase | K.TITSIAPVYLNALEK.K | Q98RA6 | 99.8 | 5.2 | 1.73 |
| 15 | ATP-dependent Zn protease | K.TMALNDANKNIIKDLMIQNK.D | Q2NJ67 | 82.9 | 7.0 | 2.85 |
| 16 | ATP-dependent protease La | K.LNFVIIDTINETEPETK.I | Q6YQC7 | 89.9 | 7.95 | 2.10 |
| 17 | molecular chaperone DnaK | R.IINEPTAAALSYGVDK.G | Q2NK65 | 66.8 | 5.17 | 2.60 |
| 18 | Heat shock 70 kDa protein (HSP70) | R.IINEPTAAALAYGIDK.T  R.MIKEAQENAANDEAKK.K | Q2SSB0 | 63.9 | 4.9 | 5.41 |
| 19 | Ffh | K.M*EEIGRM*LKANKNPFGSLF | Q7NBI0 | 50.7 | 2.7 | 7.98 |
| 20 | HPr kinase/phosphorylase | K.VFKLNPPLVILSK.S | Q6KIA8 | 36.4 | 7.8 | 4.04 |
| 21 | ATP-dependent serine proteinase - heat shock protein | R.INSILKEVK.K | Q9PQ99 | 80.6 | 6.37 | 1.28 |
| 22 | Cell division protein ftsH-like protein | K.LSEEISLEELAK.Q | Q2NIN5 | 75.8 | 5.81 | 1.78 |
| 23 | Lipoate-protein ligase A | K.GDLLLDNRGSFVINLGLK.R | Q3LBT8 | 17.2 | 9.64 | 12.10 |
| 24 | Hemolysin A | R.INSKLDHKQAILK.V | Q2ST62 | 31.2 | 9.1 | 4.83 |
|  | **Energy metabolism** |  |  |  |  |  |
| 25 | Lipoate-protein ligase A | K.EDNLASFCHAFQGVIYQTK.H | Q3LBU0 | 21.4 | 6.6 | 10.22 |
| 26 | Glyceraldehyde-3-phosphate dehydrogenase | K.KDLAHKHIEAGAK.K | A8R279 | 36.7 | 7.7 | 3.87 |
| 27 | L-lactate dehydrogenase (L-LDH) | K.M*LAAKFNTSPDAIR.A | A5IYS9 | 34.9 | 6.1 | 4.33 |
| 28 | ATP synthase subunit beta 2 | K.IGIFGGAGVGK.T; | Q98QB6 | 52.7 | 5.7 | 2.35 |
| 29 | ATP synthase alpha chain | K.LSM*LDYELNK.E | A5IY82 | 58.9 | 8.8 | 1.91 |
| 30 | Phosphoglycerate kinase | K.LITDLNLNDKKVLIR.L | Q98QW4; | 86.7 | 4.6 | 1.95 |
| 31 | Acetate kinase | R.FLSSNEGM'GATFVLADIGK.L | B3QZW9 | 43.9 | 8.41 | 4.90% |
| 32 | Pyruvate kinase | K.PVVVATQMLESMQR.N | Q2NJ42 | 50.4 | 6.31 | 3.14% |
| 33 | Pyruvate dehydrogenase E1 component subunit beta | K.LVEAEGISVEIIDLR.T | P35488 | 35.7 | 6.0 | 4.59 |
| 34 | Triosephosphate isomerase | K.M*NKTVHETRDFIQK.F | P48779 | 27.0 | 8.4 | 5.79 |
| 35 | Pentitol phosphotransferase enzyme II, B component | -.MSQQLKIIAACGNGMGTSM*IIK.L | Q98PX4 | 10.3 | 9.4 | 22.92 |
| 36 | Fructose-bisphosphate aldolase | K.AGEISKKPLIIQLSLGGIR.Y | Q4A7F3 | 41.4 | 7.7 | 6.88 |
| 37 | Phosphopantetheine adenylyltransferase | K.DFSNVEIIINENK.L | P45616 | 16.3 | 8.9 | 9.28 |
| 38 | Phosphoglycero mutase | R.EMIAYKALLGVSK.N | P47669 | 56.9 | 6.4 | 2.56 |
|  | **nucleosides and Nucleotides metabolism** |  |  |  |  |  |
| 39 | GTP pyrophosphokinase | R.EESIAKIITNIK.S | Q2NIN0 | 86.0 | 9.39 | 1.61 |
| 40 | Uridylate kinase | R.VMTAFSVPTVAEPYIRR.R | B3QZN4 | 27.0 | 9.34 | 7.10 |
| 41 | Adenylate kinase | K.ILLIGPPGSGK.G | Q9PQP0 | 24.3 | 7.7 | 5.16 |
| 42 | Adenylosuccinate lyase | K.LDMNVDSLSTQVCQRDR.H | Q2SR97 | 50.7 | 6.4 | 3.94 |
|  | **Replication** |  |  |  |  |  |
| 43 | Rep | R.NSKLREM*DK.K | Q1WM07 | 44.1 | 9.0 | 2.40 |
| 44 | Putative transposase | K.GIIQNANLR.Y | Q4A5T7 | 53.9 | 9.7 | 1.96 |
| 45 | Exopolyphosphatase-related protein | K.DSIKAFETKSQDK.N | Q6YPH9 | 36.5 | 8.0 | 4.04 |
| 46 | DNA recombination protein | K.EINENNAKSFNDIKEK.I | A5IY37 | 55.5 | 6.3 | 3.35 |
| 47 | Putative partial transposase | K.ALVEIFDSNQPR.Q | O50370 | 27.7 | 10.1 | 5.15 |
| 48 | Topoisomerase IV subunit A | -.M*NKSLDSVINSQLEK.I | Q4AAS0 | 97.1 | 9.1 | 1.77 |
| 49 | Putative DNA polymerase III | K.NFDGMVGQEVVIKTLKNAIK.L | B3R066 | 66.8 | 8.7 | 3.45 |
| 50 | DNA gyrase subunit A | K.AILEM*SLQRLSNQETQK.L | Q2NJM2 | 94.2 | 9.29 | 2.04 |
| 51 | DNA gyrase subunit B | K.TFCFDGGIVDYVKELNKGK.K | B3PND3 | 59.0 | 8.4 | 3.57 |
| 52 | DNA gyrase subunit B | K.FLNQSKSKIHNDIIYIDK.E | P05652 | 70.8 | 5.6 | 2.85 |
| 53 | DNA polymerase III polC-type | K.GDGHNIPFATFMGFAGDK.I | P75080 | 165.1 | 7.0 | 1.25 |
| 54 | DNA polymerase III alpha subunit | K.GIDPLKAFDIM*EFIR.K! | Q2NJG3 | 182.4 | 9.0 | 0.95 |
| 55 | Replication associated protein | K.INIFDDISIPQINK.E | Q0QLC1 | 45.0 | 9.1 | 3.71 |
| 56 | DNA topoisomerase IV subunit A | K.KEIEPSIEKVEEK.T | Q8EVB9 | 171.1 | 3.9 | 0.88 |
| 57 | Restriction-modification enzyme subunit R1 | K.ESIEAFLDFEIK.M | Q98PP3 | 117.2 | 8.8 | 1.22 |
| 58 | ATP-dependent DNA helicase | K.NVFIVGLSEYIWPNIK.S | Q6KHF3 | 86.9 | 8.4 | 2.16 |
| 59 | DnaG | -.M*EIICMSNDLNQLAK.Y | Q7NB89 | 78.3 | 8.3 | 2.25 |
| 60 | Foramidopyrimidine DNA glycosylase | R.M*TGKYFTDSSINRTR.K | A5IYA4 | 32.3 | 9.1 | 5.38 |
| 61 | UvrABC system protein B | R.MGKFDVVVGINLLK.E | Q9ZB21 | 75.9 | 6.0 | 2.12 |
|  | **Transcription** |  |  |  |  |  |
| 62 | RNA polymerase beta subunit | R.VPNGGDGIVQAIK.R | Q2ST48 | 64.6 | 5.6 | 2.19 |
| 63 | Cmp-binding-factor | K.LADKLNLYKDQNNK.I | Q6MTB1 | 37.5 | 6.5 | 4.29 |
| 64 | RNA polymerase beta subunit | R.SNNGSAIVQSSIVKVGDKVK.A | Q6MRX6 | 65.3 | 5.1 | 3.34 |
| 65 | RNA polymerase beta subunit | K.PQTPIVGTGVEYAVAR.D | Q6F0L7 | 65.4 | 5.8 | 2.70 |
| 66 | RNA polymerase sigma factor RpoD | -.M*AKFNNKSELK.K | Q2SRY7 | 58.1 | 4.9 | 2.18 |
| 67 | RNA polymerase beta subunit | K.VKNGHSGTIIHVEILSR.E | Q4A5S7 | 66.3 | 5.7 | 2.83 |
| 68 | RNA polymerase beta subunit | R.VGSDVTPGDVLVGR.V | Q98Q23 | 65.2 | 5.4 | 2.37 |
| 69 | DNA-directed RNA polymerase | K.GDLLVDGSSFKDGEM'SLGK.N | Q4PKC8 | 65.3 | 5.9 | 3.20 |
| 70 | DNA-directed RNA polymerase subunit beta | K.LLMELFKTIK.F | Q6F0L7 | 39.1 | 4.8 | 2.81 |
| 71 | DNA-directed RNA polymerase subunit beta | K.NIDLKDYGITSDVYGK.L | Q4A7A9 | 136.7 | 8.4 | 1.32 |
| 72 | DNA-directed RNA polymerase alpha chain | K.YIVKPLEKGFGITLGNALR.R | Q9RDV6 | 36.5 | 6.4 | 5.85 |
| 73 | RNA polymerase sigma factor | K.VSSISVEEIKMENSLK.K | Q04506 | 79.3 | 9.3 | 2.37 |
| 74 | DNA-directed RNA polymerase subunit beta' | R.M*LQEAVDALFDNER.K | Q2ST47 | 141.3 | 8.0 | 1.12 |
| 75 | RNA polymerase gamma subunit | K.RSNISETSKMLDDIK.N | Q6YQW2 | 156.6 | 8.7 | 1.09 |
| 76 | Ribonuclease R | R.ANEAVAWKM*NKLK.L | Q98QL0 | 83.7 | 7.2 | 1.79 |
|  | **Translation** |  |  |  |  |  |
| 77 | Phenylalanyl-tRNA synthetase beta chain | K.NLKLMGFNFVGK.K | Q8EUJ9 | 90.2 | 5.95 | 1.50 |
| 78 | tRNA uridine 5-carboxymethylaminomethyl modification enzyme gidA | K.ANGPLYCPSIEDKVFR.F | P75221 | 68.1 | 9.1 | 2.61 |
| 79 | Glutamyl-tRNA amidotransferase subunit A | K.IISFSEASSNLANLTGVAFGNR.E | Q4A6V8 | 48.6 | 8.9 | 5.01 |
| 80 | Putative tRNA/rRNA methyltransferase | K.ENCADITSNVLK.V | Q4L3J1 | 26.0 | 9.1 | 5.15 |
| 81 | Trigger factor | K.VSKEDVDSEIEKIR.E | Q6KIK5 | 55.1 | 9.1 | 2.90 |
| 82 | Aspartyl/glutamyl-tRNA(Asn/Gln) amidotransferase subunit B | R.PM*GAPNYGTRVEIK.N | Q7NAN0 | 55.8 | 5.9 | 2.91 |
| 83 | Leucyl-tRNA synthetase | K.EPFSSLLTQGM*VLK.D | Q4A697 | 90.3 | 8.4 | 1.83 |
| 84 | Similar to single stranded RNA(DNA) processing enzyme | R.INEIIEIAVNNK.R | P75497 | 64.1 | 7.6 | 2.11 |
| 85 | Peptidyl-tRNA hydrolase | -.M*KLIVGLGNPGDEYDK.T | Q98PE2 | 21.5 | 9.1 | 8.60 |
| 86 | Tyrosyl-tRNA synthetase | K.ALFDNQLDQLSDQEKLVVFASFDK.V | Q7NBH9 | 48.5 | 8.5 | 5.71 |
| 87 | Elongation factor tu | K.EARAGDNAGLLLRGIER.S | Q4A597 | 41.5 | 5.4 | 4.52 |
| 88 | Elongation factor tu | K.VGQEVEIVGLK.P | P13927 | 43.0 | 6.2 | 2.79 |
| 89 | Elongation factor G | K.MDKVGADFAYAIETLK.Q | Q2NJ19 | 76.3 | 5.5 | 2.33 |
| 90 | Translation initiation factor IF-2 | K.IASLKSKTTFVDK.V | Q7NBZ4 | 68.1 | 6.6 | 2.11 |
| 91 | Threonyl-tRNA synthetase | K.LENQMMMLR.P | P75225 | 64.9 | 9.0 | 1.60 |
| 92 | Methionyl-tRNA synthetase | K.M*PTKIISHGWIVTPEGK.M | Q50319 | 59.9 | 8.1 | 3.34 |
| 93 | threonyl-tRNA synthetase | K.NESILSKEM*DLK.E | Q6F1T9 | 74.7 | 5.7 | 1.86 |
| 94 | Isoleucyl-tRNA synthetase | R.CTSQWFIGLDKAK.N | Q6F175 | 104.4 | 5.6 | 1.43 |
| 95 | Modification methylase, HemK family | K.NVIEYEPSIALFAPNK.G | Q2SSX7 | 31.5 | 9.0 | 5.93 |
| 96 | 30S ribosomal protein S9 | K.KVNYFGTGRR.K | Q2NIP5 | 14.5 | 10.9 | 7.69 |
| 97 | 30S ribosomal protein S6 | K.FEQLERTELAYK.V | Q6KI63 | 18.3 | 9.7 | 7.79 |
| 98 | 30S ribosomal protein S13 | R.ILGVDIPNDK.R | Q9PQN6 | 15.0 | 11.1 | 7.58 |
| 99 | 50S ribosomal protein L1 | K.VAMLQLYLTKLNELLK.A | Q2NJ12 | 25.4 | 9.61 | 8.22% |
| 100 | 50S ribosomal protein L10 | M*KEGKGIIMIK.P | Q2NJ13 | 19.8 | 9.71 | 6.21% |
| 101 | 50S ribosomal protein L17 | R.KLFNELGPK.Y | Q9PQN3 | 13.2 | 10.5 | 7.56 |
| 102 | tRNA/rRNA methyltransferase | -.MHIIMPIAFDLDPK.H | Q4A8M2 | 17.3 | 9.3 | 9.59 |
| 103 | tRNA modification GTPase | K.AHSLNKIYVPVINKK.D | A5IXJ1 | 49.8 | 5.1 | 3.37 |
|  | **Transport and binding proteins** |  |  |  |  |  |
| 104 | ABC-type amino acid transport system | K.EIDFSKPYVFGTVKIVLK.Q | Q2NJL3 | 32.6 | 8.60 | 6.19 |
| 105 | ABC transporter extracellular solute-binding protein | R.KEFM*DKQNSEK.D | Q2NK01 | 66.3 | 9.03 | 1.93 |
| 106 | ABC-type dipeptide/oligopeptide transport system, permease component | K.M*IPGDPVDAM*FEK.G | Q6YR28 | 34.8 | 9.5 | 4.22 |
| 107 | ABC-type Cobalt import ATP-binding protein | K.TDLYLKSPFQISDGQQR.K | B1V977 | 32.5 | 9.39 | 6.0 |
| 108 | Cobalt import ATP-binding protein cbiO 1 | K.VVDM*EKHLSREPEYLSGGQK.Q | Q4A5A5 | 29.7 | 6.5 | 7.52 |
| 109 | Putative ABC substrate-binding protein - iron | K.LEKINKAPNDSEK.A | Q9PRC1 | 55.2 | 9.7 | 2.77 |
| 110 | Multiple sugar ABC transporter protein P, lipoprotein | K.VDGTAMVYRGAAQKWAITTLANDPAK.L | Q98RB2 | 53.5 | 7.6 | 5.37 |
| 111 | Hypothetical ABC transporter permease component transmembrane protein | K.GLISLKDNSR.V  K.TFVGRFRM*VVLSTSLK.Q | P75613 | 164.9 | 9.0 | 1.79 |
| 112 | Phosphonate ABC transporter, substrate binding protein | R.SMILITGSASEITAIK.K | Q49410 | 42.3 | 8.3 | 4.35 |
| 113 | Putative ABC transporter ATP-binding protein | -.M*QNQKQGLKMLVLR.L | Q4A9L9 | 26.2 | 9.6 | 6.25 |
| 114 | ABC transporter homolog | K.EISMIFQDPITSLNPMFKIK.D | Q53193 | 30.1 | 8.5 | 7.58 |
| 115 | Unspecified toxin/drug ABC transporter ATP-binding and permease protein | K.FDVILLDEVFENLQSSIFYEISK.I | Q6KHU2 | 80.2 | 9.0 | 3.40 |
| 116 | ABC Transporter ATP-binding B Protein | K.FPAQM*SGGQKQRISILR.A | Q98PF0 | 34.1 | 9.6 | 5.65 |
| 117 | ABC transporter ATP-binding protein | K.ISNNTTIIISQR.V | A5IYA0 | 67.5 | 9.5 | 2.01 |
| 118 | Unknown substrate ABC transporter permease | K.KIYLMLKNSFK.N | Q6F1W1 | 162.4 | 5.5 | 0.77 |
| 119 | ABC transporter ATP-binding protein | K.LIEKYSLNLNK.L | Q7NC47 | 67.5 | 9.3 | 1.87 |
| 120 | Putative ABC transporter ATP-binding protein | K.TRNEFMDVCYELNVRNK.M | P75444 | 37.9 | 8.8 | 5.21 |
| 121 | Unknown substrate ABC transporter permease component | K.YVINEIM*KNGNEK.D | Q6F1V8 | 207.8 | 5.3 | 0.72 |
| 122 | Oligopeptide ABC transporter, permeaseprotein (OppB) | R.AVEEKYGLNQPLIIR.Y | A5IXH5 | 41.1 | 9.6 | 4.16 |
| 123 | Spermidine/putrescine import ATP-binding protein potA | K.SWFSHEKLNLNDPIDR.E | Q9PR37 | 59.8 | 8.6 | 3.08 |
| 124 | Putative ATP-binding protein | K.NFKEAVFSIKK.I | Q4A5X6 | 112.3 | 8.2 | 1.04 |
| 125 | GTP -binding protein lepa | R.DEQGQDVLLFIDNIFR.F | Q2NJE6 | 67.9 | 8.94 | 2.00 |
| 126 | GTP-binding protein | K.AMIVGTPNVGK.S | Q2NJ33 | 33.5 | 10.01 | 3.73 |
| 127 | Putative cation transporting p-type ATPase protein | R.CDRIM*IQNEILR.L | P37278 | 104.8 | 5.4 | 1.28 |
| 128 | Hypothetical ATP/GTP-binding protein | R.WSWSFDKAIER.I | Q8EVT1 | 47.4 | 8.8 | 2.70 |
| 129 | Conserved hypothetical ATP/GTP-binding protein | K.VLQEGSSESKTWIK.V | Q9PQ10 | 58.9 | 2.6 | 7.68 |
|  | **Hypothetical proteins** |  |  |  |  |  |
| 130 | Hypothetical protein MYPU_2110 | R.KNPVLLQSVDKENTNASDLK.A | Q98QZ9 | 35.9 | 5.9 | 2.45 |
| 131 | Hypothetical protein AYWB_019 | R.DNEVMQIAKQAK.E | Q2NKA7 | 43.0 | 9.20 | 3.23 |
| 132 | Hypothetical protein AYWB_184 | K.QLKQNEDKLK.Q | Q2NJU2 | 88.1 | 5.73 | 1.33 |
| 133 | Hypothetical protein AYWB_296 | K.RKNNHQVSTNHDNQTSSSR.Q | Q2NJI0 | 6.1 | 10.18 | 36.54 |
| 134 | Hypothetical protein AYWB_202 | K.INMEIYM*IM*QQIQMR.D | Q2NJS4 | 12.1 | 9.37 | 15.00 |
| 135 | Hypothetical protein AYWB_223 | K.LDYPDTIFTDDKEK.M | Q2NJQ3 | 24.8 | 8.75 | 6.70 |
| 136 | Hypothetical protein AYWB_049 | M*KKETNQGIIR.G | Q2NK77 | 12.3 | 9.74 | 10.38 |
| 137 | Hypothetical protein AYWB_631 | R.IKDAPSDLLGVFFDGGTR.T | Q2NIJ5 | 13.2 | 6.05 | 15.93 |
| 138 | Hypothetical protein | K.ENDEYFEYKK.L | A5IYK8 | 35.2 | 5.6 | 3.31 |
| 139 | Hypothetical protein MYPU_2130 | K.ENQLTEFSEHGSANNVTK.P | Q98QZ7 | 184.8 | 6.1 | 1.11 |
| 140 | Hypothetical protein | K.ESYENILKINSK.M | Q6YPL8 | 25.6 | 9.6 | 5.58 |
| 141 | Hypothetical protein MYPE5580 | R.YLYLSGTPFR.A | Q8EVK5 | 98.4 | 8.78 | 1.20 |
| 142 | Hypothetical protein MYPU_7760 | K.FKQQQLKEFFIK.V | Q98PE7 | 60.3 | 9.5 | 2.23 |
| 143 | Hypothetical protein MSC_0633 | K.GEFKTKTQEYLWLIHNLDQSK.F | Q6MSY3 | 85.1 | 8.8 | 2.80 |
| 144 | Hypothetical protein | K.TLLINTYNDIFKTIFK.T | Q5LWK0 | 38.2 | 9.86 | 4.90 |
| 145 | Hypothetical protein MSC_0963 | K.GQKVFAYKQK.D | Q6MS19 | 227.0 | 9.0 | 0.51 |
| 146 | Hypothetical protein MSC_0032 | K.ILNVNVKAILIK.L | Q6MUJ9 | 120.8 | 9.3 | 1.14 |
| 147 | Conserved hypothetical protein | K.IMQDFQKDFNNLDRGLK.V | Q4A6V5 | 21.5 | 6.2 | 8.99 |
| 148 | Hypothetical protein PAM635 | K.DVGSIPIAR.S | Q6YPU0 | 5.5 | 9.59 | 20.60 |
| 149 | Hypothetical protein | K.INKLMQKHVSNK.T | Q6YRF8 | 37.2 | 9.4 | 3.86 |
| 150 | Hypothetical protein | K.INNLTNQSQDSLTKEIK.D | Q6YQV3 | 42.1 | 8.8 | 4.71 |
| 151 | Hypothetical protein UU328 | K.IDELTIINM*LATIMSTHYLIK.L | Q9PQG3 | 37.2 | 6.7 | 6.69 |
| 152 | Conserved hypothetical protein | K.IQDLEEELNK.Y | Q4A8R8 | 61.6 | 6.2 | 1.92 |
| 153 | Conserved hypothetical protein | K.AQTDQWANSPIVTK.T | Q7NAU9 | 66.1 | 8.5 | 2.22 |
| 154 | Conserved hypothetical protein | K.DISSVGYITNTFRGEQVDNR.H | Q4A6B6 | 13.1 | 6.5 | 18.02 |
| 155 | Conserved hypothetical protein | K.KANDEVVEKLK.S | Q4A5H4 | 64.6 | 6.2 | 1.92 |
| 156 | Conserved hypothetical protein | K.KLHTEYFKNSFM*LK.E | Q4A6R0 | 25.3 | 7.7 | 6.67 |
| 157 | Hypothetical protein MYPU_5030 | K.KTNPIFFK.S | Q98Q65 | 57.8 | 9.6 | 1.63 |
| 158 | Hypothetical protein | K.LDKTNVKLDNK.L | A5IYI2 | 64.6 | 10.0 | 1.88 |
| 159 | Hypothetical protein AYWB_223 | K.LDYPDTIFTDDKEK.M | Q2NJQ3 | 24.8 | 8.8 | 6.70 |
| 160 | Conserved hypothetical protein | K.LEKQLEEKLEEK.D | A5IYI7 | 22.9 | 4.3 | 6.25 |
| 161 | Conserved hypothetical protein | K.ISEIKSDLNSKLSILSK.R | A5IYG1 | 46.5 | 9.1 | 4.2 |
| 162 | Conserved hypothetical lipoprotein | R.YDWLIDHNVK.W | Q7NAW9 | 100.5 | 5.76 | 1.11 |
| 163 | Conserved hypothetical protein | K.NFANIVINTGRSYNDK.H | A5IYT7 | 31.3 | 9.5 | 5.76 |
| 164 | Hypothetical protein MYPU_6500 | K.NINHLEIKEK.A | Q98PR9 | 140.8 | 9.3 | 0.82 |
| 165 | Hypothetical protein | K.NKICLSFEADEK.L | Q8RK98 | 40.1 | 8.4 | 3.56 |
| 166 | Conserved hypothetical protein | K.PM*FKVVCINGGVTSFLR.K | Q7NAI2 | 149.0 | 9.1 | 1.33 |
| 167 | Hypothetical protein EUBDOL_02193 | K.QDKEAPLLVALLK.G | Q839B2 | 20.5 | 5.1 | 7.26 |
| 168 | Hypothetical protein mhp017 | K.QFKTINPNKSR.R | Q602D3 | 33.0 | 9.4 | 3.82 |
| 169 | Hypothetical protein EUBDOL_00194 | K.QIQSMYM*KEAK.Q | P96613 | 60.6 | 7.9 | 2.10 |
| 170 | Hypothetical protein MCAP_0403 | K.RAVLDSQNEFK.L | Q2SS80 | 24.5 | 8.9 | 5.35 |
| 171 | Hypothetical protein MSC_0900 | K.TSNVTNM*DGM*FKNAIK.F | Q6MS79 | 34.9 | 9.7 | 5.32 |
| 172 | Hypothetical protein MCAP_0861 | K.TTESLQQIKLELK.T | Q2SR10 | 89.2 | 6.8 | 1.73 |
| 173 | Hypothetical protein MHP7448_0714 | K.TTTTLESFLKDK.E | Q5ZZZ6 | 10.0 | 8.9 | 13.95 |
| 174 | Conserved hypothetical protein | K.YAKLIYYLDYSK.F | Q4A7A6 | 94.8 | 8.8 | 1.44 |
| 175 | Conserved hypothetical protein | K.YPNVKTIDISLLK.F | Q8EWR5 | 55.2 | 5.6 | 2.58 |
| 176 | Conserved hypothetical protein | K.YSGCWERIYQAFAER.E | Q8EWZ6 | 21.0 | 7.6 | 8.47 |
| 177 | Hypothetical protein | R.AYPNDNGDTVINDTTVK.K | Q8EW45 | 95.1 | 5.8 | 1.93 |
| 178 | Hypothetical protein PAM633 | K.EIGALPK.I | Q6YPU2 | 63.1 | 8.54 | 1.2 |
| 179 | Putative uncharacterized protein | K.ALVQDKAVTLLDANTEK.T | Q2NJ81 | 90.3 | 6.81 | 6.30 |
| 180 | Putative uncharacterized protein | K.GINNPQPTFTK. | B9A9N8 | 15.8 | 9.24 | 8.48 |
| 181 | Putative uncharacterized protein | R.IGQDFNNNM. | Q2NJQ9 | 12.0 | 9.44 | 10.08 |
| 182 | Putative uncharacterized protein | K.RMSQGM'MR.A | Q6YPK9 | 9.7 | 9.42 | 12.69 |
| 183 | Hypothetical protein n-terminal truncated | R.DKGYDFTNVNGLIINK.Q | Q9PQY0 | 47.5 | 5.9 | 3.75 |
| 184 | Conserved hypothetical protein | R.FAFFKKIYEK.Y | A5IZI7 | 33.9 | 9.0 | 3.50 |
| 185 | Hypothetical protein MCAP_0265 | R.LNFKDENITKIK.E | Q2SSL2 | 56.2 | 9.4 | 2.55 |
| 186 | Hypothetical protein | R.PKWVLESEYK.D | Q1IH50 | 31.9 | 7.8 | 3.58 |
| 187 | Hypothetical protein MYPU_4140 | R.QTLVELDGIDK.P | Q98QF2 | 260.2 | 6.5 | 0.49 |
| 188 | Hypothetical protein MSC_0677 | R.YSYNGKLDYKDFLYR.I | Q6MSU2 | 20.2 | 9.5 | 8.98 |
| 189 | Conserved hypothetical protein | R.RVLTSSITQSISLK.L | Q4A858 | 227.9 | 9.0 | 0.71 |
| 190 | Unique hypothetical protein | R.QGTSASYIRSDANK.I | Q7NAW3 | 208.9 | 5.5 | 0.75 |
| 191 | Conserved hypothetical protein | K.NLKFFREDATLK.N | Q4A5I2 | 155.5 | 9.3 | 0.92 |
| 192 | hypothetical protein AYWB_205 | R.NKTRTDIDEEIK.T | Q2NJS1 | 12.0 | 9.79 | 12.12 |
|  | **Other** |  |  |  |  |  |
| 193 | Predicted hydrolase Cof | K.LIGIDLDGTLLSIR.K | Q7NAT6 | 33.5 | 9.4 | 4.78 |
| 194 | Uncharacterized protein MG075 homolog | K.AMLEFLPDTKDLETTLDK.L | P75556 | 116.0 | 5.9 | 1.75 |
| 195 | Phosphoglycolate phosphatase | -.M*FM*IILLLIIIQIRR.K | Q9PR29 | 28.8 | 7.2 | 6.10 |
| 196 | PvpA | R.PM*GAGGSNQPRPM*PNGLQK.Q | Q11PK5 | 7.4 | 12.3 | 27.14 |
| 197 | Transposase | R.NSFNLENKAVK.Q | Q8KML2 | 40.1 | 9.8 | 3.22 |
| 198 | Hypothetical transmembrane protein | R.GMYQNINGVNPIAYLYFQK.Y | Q6F163 | 122.3 | 9.2 | 1.78 |
| 199 | Vsaa-like protein | R.ENLLNLSKILFNQANR.D | Q98QQ3 | 117.4 | 5.5 | 1.57 |
| 200 | Surface protective antigen SpaA | K.YEDKVKGR.A | Q38009 | 72.4 | 8.9 | 1.28 |
| 201 | Predicted HD phosphohydrolase | K.NNVVKSLTK.F | Q6F0G7 | 47.1 | 7.3 | 2.24 |
| 202 | Protoporphyrinogen oxidase | K.LKEEISEKELK.M | Q6KIA1 | 27.6 | 6.6 | 4.66 |
| 203 | PepB | K.KM*GLNLLYGVNKGSER.E | Q7NB27 | 49.4 | 8.8 | 3.59 |
| 204 | Expressed protein | K.FISESIMQVNKK.F | Q6KHR1 | 46.1 | 6.6 | 3.03 |
| 205 | Hypothetical trag protein n-terminal truncated | K.INNIEKWVSISR.S | Q14Q74 | 41.8 | 8.8 | 3.39 |
| 206 | Putative type I restriction enzyme specificity protein MPN201 | K.LCNDLVEGIPAEIELR.K | Q50287 | 27.5 | 8.4 | 6.72 |
| 207 | Prolipoprotein | R.SFIDKPGLTDAFLAANK.V | Q6MSZ1 | 98.4 | 8.7 | 1.98 |
| 208 | Lipoprotein | K.TTQVNKTKTDTELR.E | Q98RF3 | 16.3 | 9.6 | 10.00 |
| 209 | Lipoprotein | K.VFFNGFIDKIVLSGMLR.P | Q4A7L8 | 107.4 | 8.9 | 1.81 |
